# Using plant electrical signals of water hyacinth (*Eichhornia crassipes*) for water pollution monitoring

**DOI:** 10.1515/biol-2025-1120

**Published:** 2025-10-18

**Authors:** Valeria Maria Melleiro Gimenez, Ana Carolina de Souza Silva, Gustavo Maia Souza, Ernane Jose Xavier Costa

**Affiliations:** Department of Basic Science, FZEA, University of Sao Paulo, Rua Duque de Caxias Norte, 225, Pirassununga, 13630-090, Sao Paulo, Brazil; Department of Botanic, Federal University of Pelotas, Institute of Biology, Pelotas, 96010-610, RS, Brazil

**Keywords:** plants’ electrical signals, water monitoring, digital signal processing, interface plant-computer, bio-indicator

## Abstract

Aquatic plants, such as water hyacinths, *Eichhornia crassipes*, are indicators of environmental changes. This study explores the response of water hyacinths to wastewater exposure by analyzing their bioelectrical signals. The analysis includes time, frequency, and joint time-frequency domains, evaluating the plant’s response to water quality variation. In the time domain, the Lempel-Ziv complexity analysis was used to demonstrate how the plant’s response evolves over time, while spectral entropy was used for frequency domain analysis. By using adaptive Gabor representation, the joint time-frequency behavior of the signal was evaluated. All these advanced digital signal processing techniques were used to evaluate the plant’s ability to detect and adapt to the presence of pollutants. The results show that water hyacinths can serve as part of a reliable instrumentation system for real-time aquatic ecosystem monitoring, as the plant’s bioelectrical signals changed both in the time domain and frequency domain.

## Graphical abstract

**Figure:**
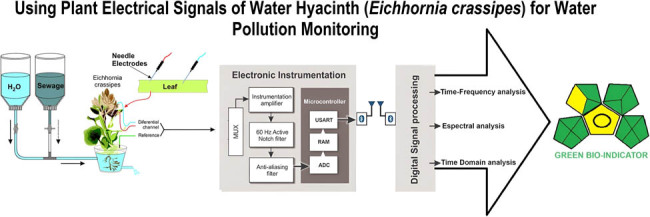

## 1 Introduction

The use of plant bioelectrical signals as a measurement system is a challenge in the field of bio-detectors, which has gained considerable attention in scientific literature. Traditional analytical methods, such as chromatography and spectroscopy, have practical limitations for real-time monitoring [1,2,3]. A long-term alternative would be to use plant bioelectricity to monitor the environment by interfacing with electronic systems and tracking their bioelectrical responses [4,5]. Currently, the monitoring of pollution sources in a water supply network based on smart electronic instrumentation methods has become the state-of-the-art approach to fulfill the need for information on water quality. This requirement arises from the necessity of ensuring a specific quality of water for various purposes. Indeed, there is a growing academic consensus that “water quality” should encompass the monitoring and investigation of the physical, chemical, and biological characteristics of water [6]. Over time, new challenges in water monitoring have emerged, such as acquiring data on the relationship between the environment and water quality and integrating the systemic constituents of water, such as aquatic plants, within the context of water monitoring [7]. The water hyacinth (“*Eichhornia crassipes*”) plays a prominent role among aquatic plants in relation to water pollution control. Among aquatic macrophytes, water hyacinth is considered a suitable species for phytoremediation and bio-remediation [8]. Under controlled growth conditions, it surpasses conventional methods by efficiently extracting and absorbing industrial and agricultural effluents contaminated with organic, inorganic, and toxic residues [9,10].

Most studies have focused on the use of water hyacinth for wastewater treatment [11]. Therefore, a systematic technique was developed to evaluate the potential of water hyacinth in improving water quality monitoring by harnessing bioelectricity [12]. The phenomenon of electrical biopotentials in plants has raised new scientific questions about plant physiology [13]. These electrical signals are generated through transient depolarization/hyperpolarization events, resulting from changes in the membrane potential induced by various stimuli. These events modulate ion channels and the voltage of the plasma membrane, causing an imbalance of ions [14].

Four types of electrical signals have been reported in plants: action potentials (AP), variation potentials (VP), local electrical potentials (LEP), and system potentials (SP) [15]. Among these signals, APs have been the most extensively studied. Essentially, bioelectrical activity involves the transient disruption of the ion balance (such as Ca^2+^, K^+^, and Cl^−^) across the plasma membrane, often triggered by external stimuli [16]. On the other hand, SP is a systemic hyperpolarization event that propagates from leaf to leaf, depending on the intensity and nature of the original stimulus [17], while LEP represents a sub-threshold response induced by changes in environmental factors (such as water, air temperature, and humidity, light, and fertility). LEP is generated locally and does not spread to other parts of the plant [18,19].

Plant electrical signals play significant physiological roles in nutrient absorption, respiration, photosynthesis [20], and phloem transport [21]. It has been demonstrated that not only plant activities but also the plant environment can be monitored using bioelectrical signals [22]. In aquatic plants like water hyacinths, it is well established that their roots play a crucial role in the interaction between plants and pollutants [23,24]. Therefore, it is reasonable to assume that changes in root physiology will propagate through the plant’s vascular system and subsequently affect the patterns of bioelectrical signals. Consequently, integrating bioelectrical signals from plants with digital signal processing (DSP) techniques can enhance the monitoring of water pollutants using water hyacinth, coupled with an integrated electronic signal acquisition and processing system.

Advancements in DSP have equipped researchers with robust tools to analyze the intricate bioelectrical signals in plants. Spectral entropy, introduced by Inouye et al. [25], has been effectively utilized to quantify the complexity of time-series data, including bioelectric signals, by measuring the disorder or unpredictability within the signal. Similarly, Lempel-Ziv complexity, initially developed as a data compression algorithm, serves as a metric for assessing the irregularity and unpredictability of time-series data. Its application in analyzing physiological signals, such as electroencephalograms, has demonstrated its efficacy in capturing the complexity inherent in biological systems [25,26]. The adaptive Gabor transform is a powerful means to analyze the time-frequency characteristics of non-stationary signals [27]. By providing a joint time-frequency representation, it facilitates the detection of transient features and dynamic changes within the signal. In the realm of biological signal analysis, the adaptive Gabor transform has shown promise in elucidating the temporal and spectral components of plant bioelectrical activity. Integrating these advanced DSP techniques into an electronic signal acquisition and processing system enables real-time and continuous monitoring of water pollutants through the bioelectrical responses of water hyacinths. This approach leverages the inherent sensitivity of plants to environmental changes, enhancing our understanding of plant–environment interactions and offering a novel method for environmental monitoring [28]. In the context of monitoring water pollutants using aquatic plants like water hyacinths, these DSP techniques can play a pivotal role. Spectral entropy can capture changes in the complexity of bioelectrical signals as the plants respond to varying pollutant concentrations and environmental conditions. Lempel-Ziv complexity can provide insights into the unpredictability of the bioelectric signals, potentially indicating stress or perturbations in the plant’s physiological state. Adaptive Gabor transform, with its ability to analyze time-frequency patterns, can unveil dynamic changes in bioelectrical signal patterns that might correlate with pollutant exposure events. Coupling the inherent sensitivity of water hyacinths to pollutants with advanced DSP techniques not only enhances our understanding of plant–environment interactions but also offers a novel approach to environmental monitoring. This integrated approach aligns with the principles of green analytical chemistry (GAC), which encourages the reduction of toxic chemicals, the use of energy-efficient equipment, and the generation of minimal waste [29].

The objective of this article is to demonstrate, under laboratory conditions, that the bioelectrical response of water hyacinth differs when exposed to sewage water compared to clean water. This study investigates the bioelectrical activity of water hyacinths under controlled conditions, utilizing advanced DSP techniques to assess their response to sewage water exposure. The research explores the viability of employing aquatic plants, specifically water hyacinth, as bio-detectors for water monitoring through precise DSP methodologies.

## 2 Botanic material: Description and cultivation

**Figure 1:**
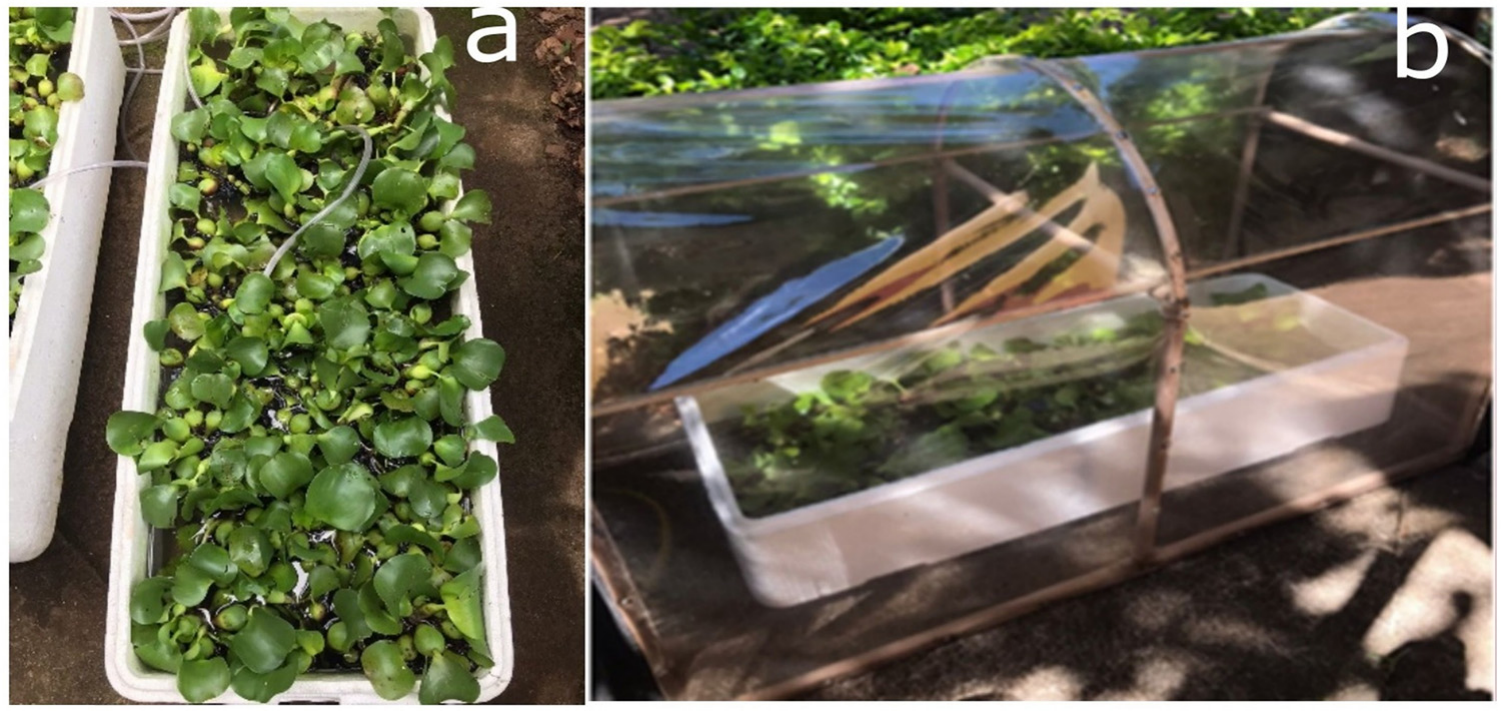
Water hyacinth tank box with protection and aeration.

Water hyacinth samples were collected from water catchment ponds on the University of São Paulo campus in Pirassununga. These samples were transferred to thermally insulated tank boxes (made of Styrofoam), measuring 1 m × 0.40 m × 0.25 m and with a capacity of 100 L. The samples were placed outdoors under natural light for a 30-day acclimatization period. A protective covering was provided using polyvinyl chloride (PVC) tubes covered with transparent plastic and shade netting (Figure 1).

Physicochemical monitoring of the tank boxes was conducted, including pH control within the range of 5.5–6.5, turbidity measurement using a Hanna HI98703 device, as well as monitoring light and temperature.

The analyte used was raw urban sewage, collected from the Water and Sewage Treatment Station of the municipality of Pirassununga. The collected material was placed in plastic containers and maintained at a controlled temperature of 22°C for a maximum of 6 h during each experiment.

## 3 Experimental setup

**Figure 2:**
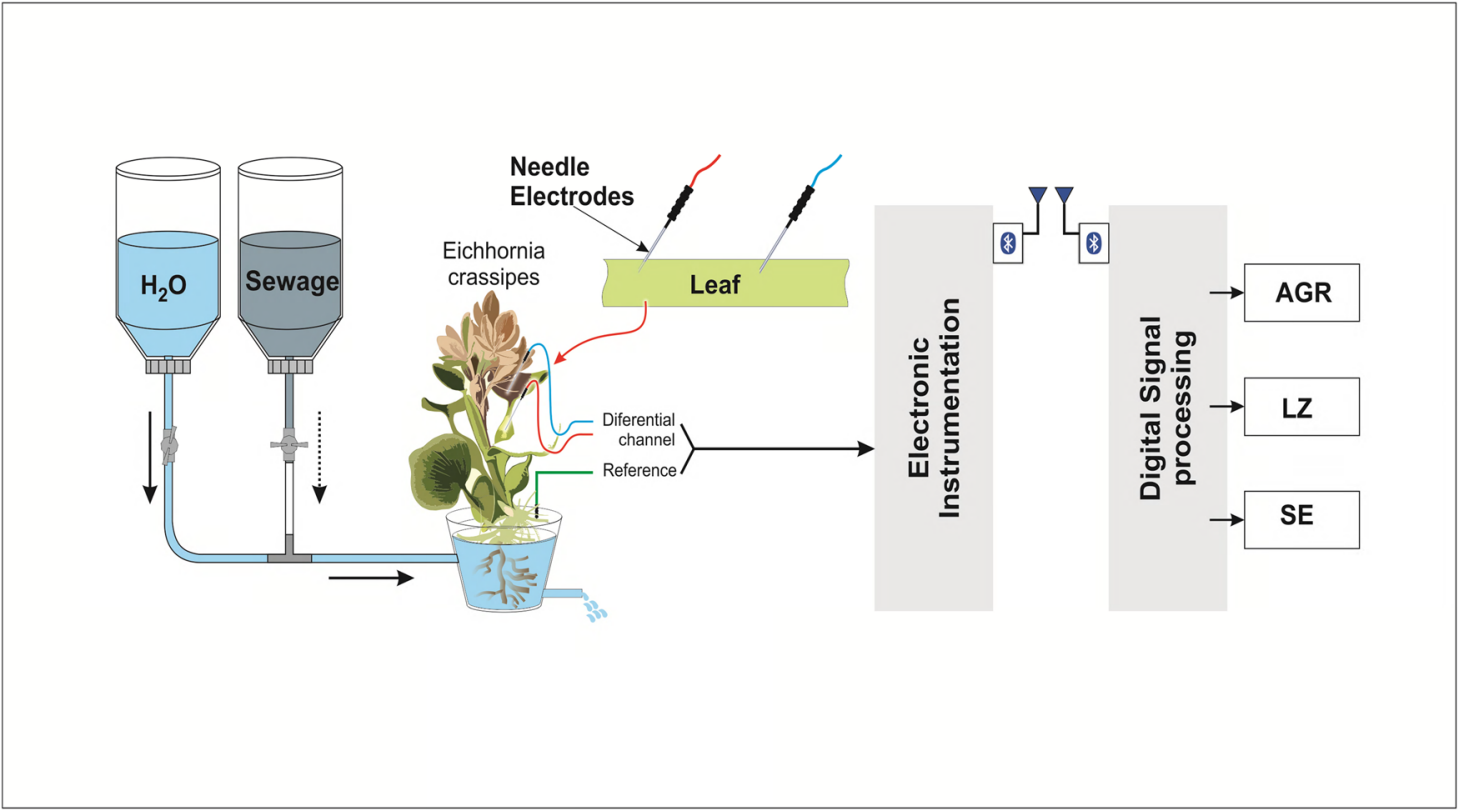
Experimental setup.

From the cultivated water hyacinth plants, 30 most vigorous and robust adult specimens were randomly selected from a larger population of healthy plants and used for the proposed experiments. This approach minimizes selection bias and enhances the generalizability of the findings. Each of these plants was individually placed in an experimental unit composed of a container with a capacity of 1 L of nutrient solution, along with a silicone hose and a flow control device (Figure 2).

The bioelectrical signals were monitored using two Ag/AgCl needle electrodes positioned on one of the plant’s leaves, maintaining a distance of 5 cm between them. A ground electrode was connected to the container system. To ensure stable experimental conditions and minimize external interference, each plant remained inside a Faraday cage, an electromagnetically isolated environment, at an average temperature of 23°C and a light intensity of 100 lux for 1 h. After this adaptation period, the bioelectric potentials began to be recorded. The experimental setup also included a 5-L reservoir directly attached above the experimental unit. This reservoir worked in conjunction with a gravity drainage system, providing a flow rate of 1 L of solution at intervals of 3 min. The transfer of this solution was carried out through a silicone hose connecting the reservoir to the experimental unit. Additionally, another container of identical capacity was incorporated just below the experimental unit, specifically designed to collect and direct the flow of released solutions. The bioelectrical signals (bioelectric potentials) were recorded using Ag/AgCl needle electrodes and an electronic device designed for data acquisition. Two minutes after the start of the plant’s biopotential recording in the culture medium, a flow of 2 L of sewage from container 1 begins, and completely replaces the culture medium in the experimental unit. At the same time, the culture medium is gradually transferred to vessel 2. Thus, in 18 min, it was possible to record the plant’s biopotentials under different conditions: in its culture medium, when the analyte entered the experimental unit, and in the complete replacement of the medium by the analyte.

### 3.1 Acquisition of bioelectric signals

The bioelectrical signal acquisition system employed in this study was adapted from the design proposed by Cabral [30]. The original system utilized an instrumentation amplifier with four inputs to operational amplifiers, designed to pre-amplify the signal, apply both high-pass (cutoff frequency of 0.5 Hz) and low-pass (cutoff frequency of 1.5 kHz) filters, and subsequently post-amplify the signal to ensure a clean and robust output. The digitization was performed using a bipolar 10-bit analog-to-digital converter, with a microprocessor controlling the signal sampling frequency and data transmission via an RS232 interface. In our implementation, we employed the ATmega328P [31] microcontroller to manage the signal sampling at a frequency of 100 Hz and facilitate wireless data transmission using the Bluetooth protocol. The choice of a 100 Hz sampling rate was deliberate to effectively capture the physiological dynamics of plant bioelectrical signals, which predominantly occur below 50 Hz. This rate ensures adequate temporal resolution to detect rapid signal variations while mitigating potential aliasing effects. Lower sampling frequencies might overlook these swift transients and be more susceptible to low-frequency noise interferences, such as thermal fluctuations and environmental variations, which could obscure the bioelectrical signals of interest.

### 3.2 Signal processing and data analysis

The recorded bioelectrical signals were processed using Python-based algorithms. Three primary signal metrics were analyzed.

#### 3.2.1 Adaptive Gabor representation (AGR)

This technique captured time-frequency coefficients, enabling the characterization of dynamic spectral changes in the signals. Coefficients were computed over non-overlapping 2-min windows to observe temporal variations in response to analyte exposure. For each non-overlapping 2-min window, time-frequency coefficients were calculated using the AGT, resulting in a representation of the signal in the time-frequency domain. Mathematically, the AGT involves the calculation of time-frequency coefficients *G*(*t*, *f*) by convolving the signal *x*(*t*) with a set of adaptive Gabor filters. These coefficients are obtained as follows (1):

(1) \[G(t,\text{}f)\hspace{.25em}=\hspace{.25em}x(t)\times g(t,\text{}f),]\]

where *G*(*t*, *f*) represents the calculated time-frequency coefficient, *x*(*t*) is the original signal, and *g*(*t*, *f*) denotes the adaptive Gabor filter. The coefficients *G*(*t*, *f*) collectively form the AGR for the given signal segment.

Spectral entropy: Shannon entropy of the power spectral density (PSD) quantifies the complexity of the signals. PSD was estimated using Welch’s method, and entropy was calculated for each 2 min segment to assess frequency-domain variations. Mathematically, this can be expressed as follows (2):

(2) \[H(x,{f}_{s})=-\mathop{\sum }\limits_{f=0}^{\frac{{f}_{s}}{2}}P(f){\log }_{2}{[}P(f)],]\]

where *H*(*x*, *f*_s_) represents the spectral entropy, *P*(*f*) denotes the normalized PSD, and *f*_s_ corresponds to the sampling frequency. To calculate *P*(*f*), Welch’s method was employed, which estimates PSD by partitioning the signal into segments, computing periodograms for each segment, and then averaging the resulting periodograms. A 95% confidence interval was considered in the estimation process.

#### 3.2.2 Lempel-Ziv complexity

This metric evaluated the irregularity and unpredictability of the signals by analyzing binary representations of the data. Complexity values were computed for each 2-min window, providing insights into signal anomalies or stress responses. For each non-overlapping 2-mine window, the binary representation of the signal was analyzed using the LempelZiv algorithm, resulting in the complexity value (3):

(3) \[{\mathrm{LZC}}(x)=\frac{C(x)}{N(x)},]\]

where LZC(*x*) represents the LempelZiv complexity of the signal segment *x*, *C*(*x*) denotes the compressed length, and *N*(*x*) corresponds to the original length of the data after conversion to a binary sequence. The analysis facilitated by LZC provided insights into the patterns and anomalies present in the bioelectrical signals.

### 3.3 Statistical analysis

Statistical analysis was conducted in two stages. Initially, the results were presented as the mean and standard error of the mean. Subsequently, the bootstrap statistical technique was employed to provide a deeper analysis of the data. The bootstrap method was selected because it enables the estimation of a statistic’s distribution (e.g., the mean) by resampling with replacement from the original dataset. This approach allows inferences to be made about the general behavior of plants as bioindicators.

The bootstrap process involves generating a large number (typically thousands) of resampled datasets, each of the same size as the original dataset. For each resampled dataset, the statistic of interest is calculated, and the resulting distribution of resampled statistics is used to derive the standard error and confidence intervals. This methodology is particularly beneficial in ecological studies with limited sample sizes, as it provides a robust framework for estimating the variability and reliability of observed effects.

The bootstrap algorithm can be summarized as follows:

1. From a sample of size *n*, generate a new sample by randomly drawing observations with replacement from the original dataset.
2. Calculate the statistic of interest (e.g., mean, variance) for the resampled dataset.
3. Repeat steps 1 and 2 a large number of times (e.g., 1,000 or 10,000 iterations).
4. Use the distribution of the resampled statistics to calculate the standard error, bias, and confidence intervals.

The experimental design accounted for minimizing natural environmental variations by conducting experiments under controlled conditions. The use of spectral entropy, LZC, and AGR ensures that the observed signal variations are specific to wastewater exposure, as these methods are sensitive to changes in signal irregularity and complexity induced by external stressors.

## 4 Results and discussion

### 4.1 Bioelectrical signal response

**Figure 3:**
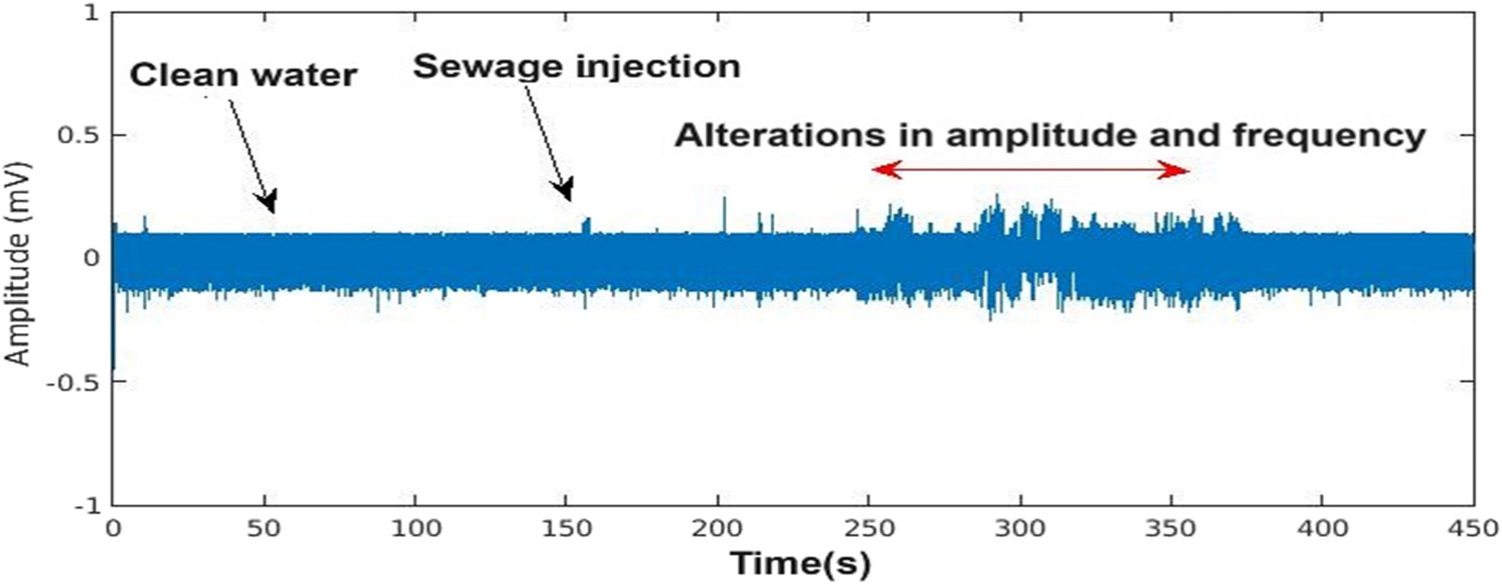
Time-domain variations in the bioelectrical signal.

The bioelectrical signals recorded from water hyacinths under different conditions (nutrient solution, sewage introduction, and complete sewage replacement) exhibited distinct variations. In the time domain, abrupt changes in the signal amplitude were observed when sewage was introduced, indicating an immediate physiological response to the altered environment. This aligns with previous studies suggesting that external stressors trigger measurable bioelectrical changes in plants [14,16]. Figure 3 illustrates the time-domain variation in bioelectrical signals during the three phases of the experiment. The signals displayed a significant increase in amplitude within the first 4 min of sewage introduction, stabilizing once the medium was fully replaced.

This distinct change occurred precisely when the wastewater began flowing through the water hyacinth vessel. However, this temporal representation does not allow for quantifying the signal’s frequency behavior or other subtle variations in the time domain, such as signal complexity.

### 4.2 Time-domain analysis

**Figure 4:**
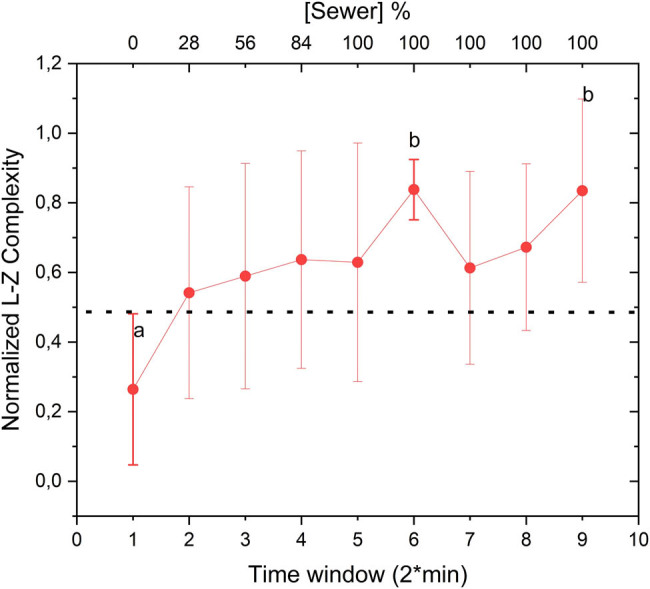
Temporal variation of LempelZiv complexity coefficients, with “a” and “b” indicating statistically distinct points based on error bars.

Lempel–Ziv analysis revealed a marked increase in signal complexity during the sewage replacement phase (Figure 4). This increase corresponds to the plant’s adaptive response to the pollutants, as the LempelZiv algorithm captures changes in the regularity of the signal in the time domain. These results are consistent with the hypothesis that stress-induced physiological changes in plants are reflected in the complexity of their bioelectrical activity [25]. Each coefficient represents the average of 30 measurements, with error bars indicating the standard error of the mean to highlight statistical differences. Normalized coefficients within the 2-min window prior to wastewater exposure (labeled as “a”) are statistically distinct, as evidenced by the error bars, from those calculated after 100% of wastewater had flowed through the roots (labeled as “b”). A notable trend emerged, showing that coefficients began to increase significantly after just 4 min, corresponding to 28% of wastewater exposure.

**Figure 5:**
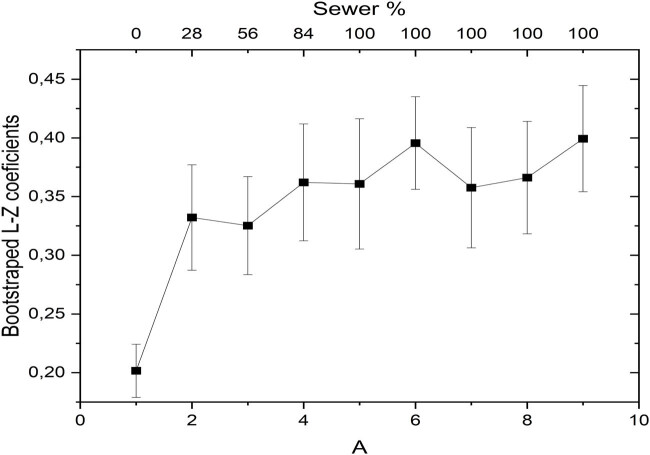
Bootstrap analysis confirming statistical significance of LempelZiv complexity trends during wastewater exposure.

To confirm the statistical validity of this observed trend, a bootstrap analysis was performed, as illustrated in Figure 5. The bootstrap method was utilized to evaluate the statistical behavior of the mean trend by generating resampled datasets and recalculating the coefficients. Starting from the 2-min window (corresponding to 28% wastewater exposure), the analysis revealed significant differences in the coefficients, reinforcing the trend within the calculated error margins. These error margins represent the standard deviation of the mean coefficients derived from all plants included in the experiment. Collectively, these results emphasize how the complexity metric effectively captured the temporal behavior of the bioelectrical signals observed in Figure 3, where the time-domain signal demonstrated distinct responses to wastewater exposure.

### 4.3 Frequency-domain analysis

**Figure 6:**
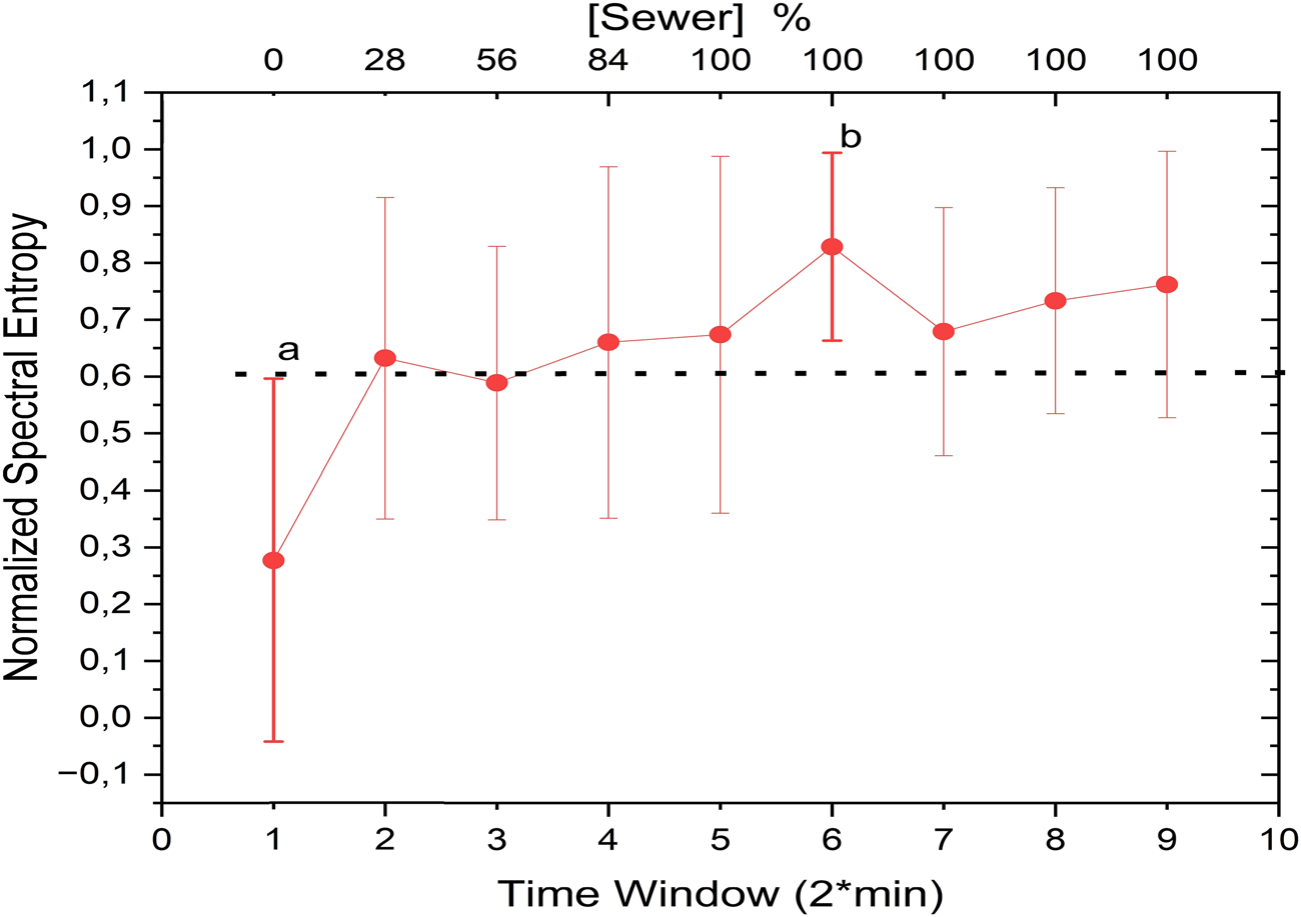
Temporal variations in spectral entropy where “a” and “b” denote statistically distinct values correlating with pre- and post-exposure conditions.

Spectral entropy analysis revealed variations in signal complexity during the sewage replacement phase in the frequency domain (Figure 6), a finding that is consistent with previous reports in the literature. Recent studies, such as Gómez Acosta and Chacón Pacheco [26], have emphasized the sensitivity of spectral entropy to physiological signal irregularities and its potential for detecting subtle changes in bioelectrical patterns, supporting its applicability to environmental signal analysis. Notably, the spectral entropy coefficients exhibited changes at the same pollutant concentrations observed in the LempelZiv complexity coefficients, as shown in Figure 5. These similarities indicate that the spectral entropy metric captured the same temporal behavior reflected in the complexity analysis, further strengthening the robustness of the observed trend.

The increase in spectral entropy during sewage exposure corresponds to the plant’s adaptive response to pollutants, capturing irregularities in signal patterns within the frequency domain. These results mirror the findings in the time domain (Figure 3), where significant changes in the signal amplitude were observed. To validate these trends, bootstrap analysis was also performed on the spectral entropy coefficients, confirming the statistical reliability of the observed variations. This additional validation underscores the utility of spectral entropy in characterizing the plant’s physiological responses to environmental stressors.

**Figure 7:**
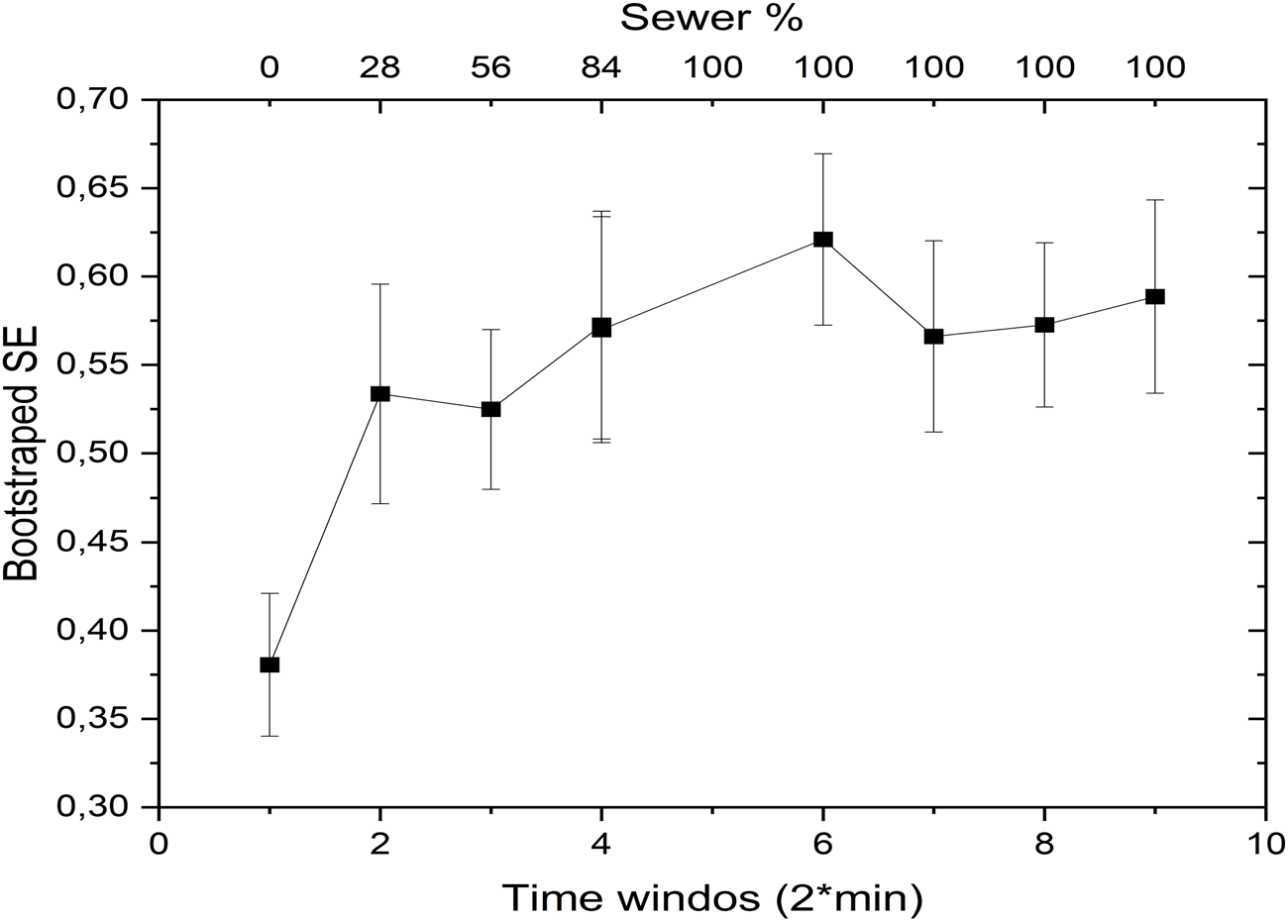
Bootstrap analysis of spectral entropy.

The results of the bootstrap analysis applied to spectral entropy are shown in Figure 7.

### 4.4 Time-frequency analysis

**Figure 8:**
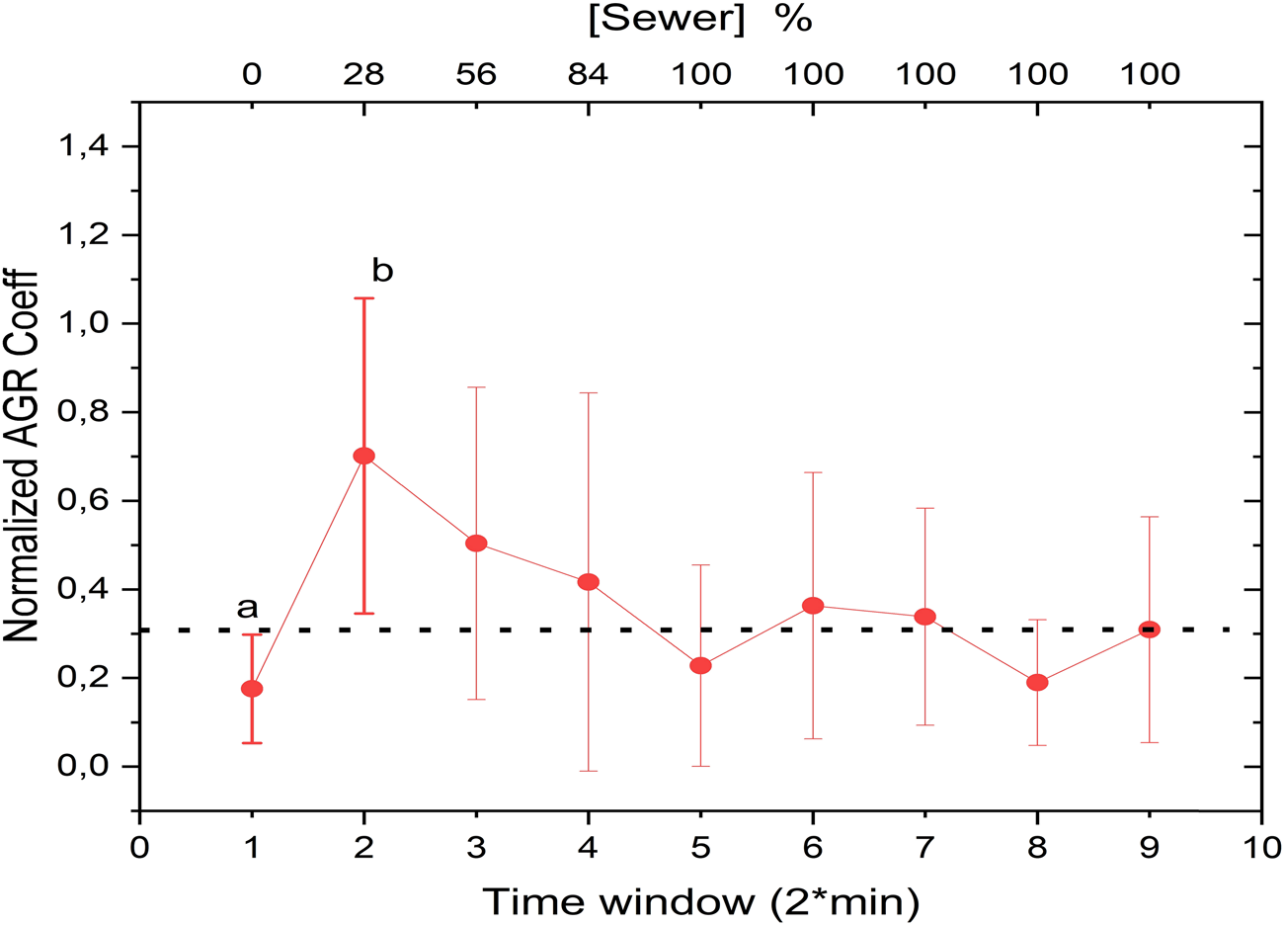
Temporal variations in normalized AGR coefficients with “a” and “b” indicating statistically distinct points representing pre- and post-exposure conditions.

The investigation into the AGR coefficients, which combine both time and frequency domains, yielded intriguing observations. Figure 8 displays how the AGR coefficients vary when the water hyacinth is exposed to 28% of wastewater. Notably, these coefficients rapidly revert to their baseline values within 5–10 min following wastewater exposure. This behavior suggests that, in the joint time-frequency domain, the plant initially senses the presence of wastewater but later adapts, eventually reverting to its pre-exposure state. These findings align with the time-domain observations shown in Figure 3, where the amplitude of the signal also returned to baseline levels after adaptation.

**Figure 9:**
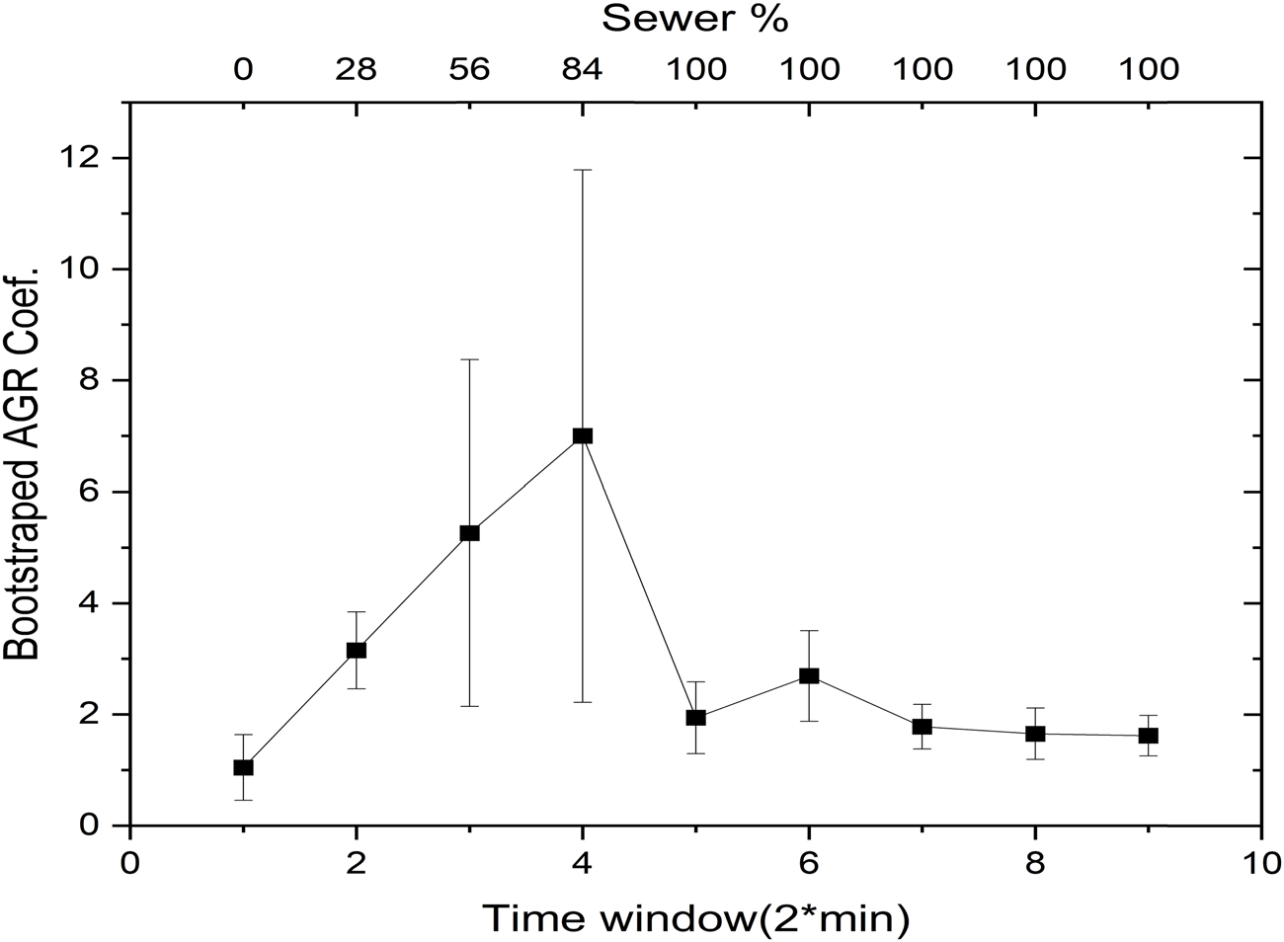
Bootstrap analysis of normalized AGR coefficients.

To confirm the statistical validity of the AGR trends, bootstrap analysis was conducted on the AGR coefficients (Figure 9). This validation confirmed the robustness of the observed patterns, providing additional confidence in the results.

Interestingly, the time-frequency domain analysis may better capture the plant’s stimulus-response behavior compared to the time- or frequency-domain methods. This could be attributed to the AGR algorithm’s ability to capture localized changes in the signal, as opposed to the other two techniques, which are more suited to detecting broader-scale changes. The number of points analyzed could reasonably explain the observed variations among the methods used. Both spectral entropy and LempelZiv complexity measurements are more sensitive to the number of points analyzed, achieving greater precision when the dataset is sufficiently large. In contrast, the AGR method appears to capture localized changes effectively, making it less dependent on the number of points and potentially explaining the difference observed.

The effectiveness of AGR in capturing dynamic changes in the bioelectrical signals of water hyacinth aligns with previous studies that have applied Gabor transforms to non-stationary biological signals, particularly in the low-frequency range, highlighting its capability to provide detailed time-frequency representations [32].

These observations highlight the potential of AGR for practical applications, where a more precise understanding of localized plant responses to environmental stimuli is critical. Our findings also corroborate previous research that has utilized bioelectrical signals to monitor the physiological responses of plants to environmental stimuli. For example, Volkov and Markin [12] demonstrated that electrical signals in plants can serve as reliable indicators of responses to various environmental stresses, reinforcing the potential of bioelectricity as a monitoring tool. By integrating time-frequency analysis, the study advances the potential for water hyacinths as bio-sensors in real-world monitoring scenarios. Also, while the experimental design prioritizes eco-friendly practices, the use of wastewater in a controlled laboratory setup raises questions about potential environmental impacts. Proper disposal of wastewater post-experiment was ensured to mitigate contamination risks, aligning with the principles of GAC. Future research should explore the reproducibility of these findings under diverse environmental conditions, such as varying temperatures, pollutant concentrations, and light intensities. This would validate the robustness of the observed trends and expand the applicability of this approach to real-world scenarios. Compared to conventional water quality monitoring techniques, such as chemical assays and spectrometric analyses, the integration of bioelectrical signals and advanced DSP offers a cost-effective, real-time alternative. However, traditional methods may provide higher specificity for certain pollutants, underscoring the need for a hybrid approach in comprehensive monitoring systems.

## 5 Conclusion

In this study, we employed a multifaceted approach to investigate the response of water hyacinths to wastewater exposure through bioelectrical signal analysis. By combining bioelectrical signal measurements with advanced DSP techniques, the research highlights a novel approach to real-time environmental monitoring. The results presented here provide strong evidence that water hyacinths respond sensitively to wastewater exposure, with measurable variations in bioelectrical signal amplitude, complexity, and frequency characteristics.

In the time domain, abrupt changes in the signal amplitude upon wastewater exposure underscore the plant’s ability to detect environmental stressors rapidly. In the frequency domain, spectral entropy captured variations consistent with those observed in LempelZiv complexity, demonstrating that both metrics effectively characterize the plant’s adaptive response to pollutants. However, the time-frequency domain analysis, utilizing AGR, emerged as a particularly promising method, capturing localized and transient changes in bioelectrical signals. This adaptability to both short-term responses and recovery behaviors suggests AGR may be especially suitable for practical applications requiring precise and dynamic monitoring. The findings also reveal methodological insights: while spectral entropy and Lempel-Ziv complexity depend heavily on the number of points analyzed, AGR’s localized focus offers a complementary perspective, making it less sensitive to dataset size. This distinction could be pivotal in tailoring monitoring systems for specific environmental contexts. Despite the promising results, this study has limitations. The experiments were conducted under controlled laboratory conditions, which may not fully replicate the complexity of natural aquatic ecosystems. Future research should validate these findings in field settings, examining the reproducibility and scalability of this approach over extended monitoring periods and across diverse environmental conditions. Although this study demonstrates the potential of water hyacinths for real-time monitoring, the long-term stability and adaptability of this approach remain unexplored. Addressing this limitation would provide insights into the feasibility of continuous monitoring over extended periods.

In conclusion, integrating water hyacinths as bioindicators in pollutant detection systems holds significant potential for practical applications, particularly in remote areas where conventional monitoring infrastructure is limited. However, to enhance the system’s robustness under environmental conditions that differ from controlled laboratory settings, *in situ* studies are essential. Field research will provide valuable insights into the performance and reliability of water hyacinth-based detection systems, ensuring their effectiveness in diverse and challenging environments. Such studies are crucial for developing sustainable and efficient water quality monitoring solutions tailored to the specific needs of remote communities. This approach aligns with the principles of GAC, emphasizing minimal environmental impact. With further refinement, this system has the potential to improve water monitoring practices, combining the sensitivity of biological systems with the analytical power of digital technology.
